# Detection and Mitigation of RPL Rank and Version Number Attacks in the Internet of Things: SRPL-RP

**DOI:** 10.3390/s20215997

**Published:** 2020-10-22

**Authors:** Zahrah A. Almusaylim, NZ Jhanjhi, Abdulaziz Alhumam

**Affiliations:** 1 Department of Computer Science, College of Computer Science & IT, King Faisal University, 31982 Al-Hasa, Saudi Arabia; aahumam@kfu.edu.sa; 2 School of Computer Science and Engineering (SCE), Taylor’s University, Lakeside Campus, Subang Jaya 47500, Malaysia; noorzaman.jhanjhi@taylors.edu.my

**Keywords:** IoT, security, RPL, rank attack, version number attack

## Abstract

The rapid growth of the Internet of Things (IoT) and the massive propagation of wireless technologies has revealed recent opportunities for development in various domains of real life, such as smart cities and E-Health applications. A slight defense against different forms of attack is offered for the current secure and lightweight Routing Protocol for Low Power and Lossy Networks (RPL) of IoT resource-constrained devices. Data packets are highly likely to be exposed in transmission during data packet routing. The RPL rank and version number attacks, which are two forms of RPL attacks, can have critical consequences for RPL networks. The studies conducted on these attacks have several security defects and performance shortcomings. In this research, we propose a Secure RPL Routing Protocol (SRPL-RP) for rank and version number attacks. This mainly detects, mitigates, and isolates attacks in RPL networks. The detection is based on a comparison of the rank strategy. The mitigation uses threshold and attack status tables, and the isolation adds them to a blacklist table and alerts nodes to skip them. SRPL-RP supports diverse types of network topologies and is comprehensively analyzed with multiple studies, such as Standard RPL with Attacks, Sink-Based Intrusion Detection Systems (SBIDS), and RPL+Shield. The analysis results showed that the SRPL-RP achieved significant improvements with a Packet Delivery Ratio (PDR) of 98.48%, a control message value of 991 packets/s, and an average energy consumption of 1231.75 joules. SRPL-RP provided a better accuracy rate of 98.30% under the attacks.

## 1. Introduction

The Internet of Things (IoT) is the consequence of the seamless integration of devices between wireless communications and diverse technologies. The devices can perceive their surrounding environment and gather data from it for processing and decision making [1]. The smart city is one of the substantial domains of IoT. A smart city is composed of innumerable services and applications that aim to increase the quality of life and services to residents [2,3,4]. However, the devices must communicate via the network layer in the IoT architecture. This layer utilizes various standards, protocols, and techniques, such as Routing Protocol for Low Power and Lossy Networks (RPL), protocols to smoothen the secure transfer of data packets between devices. The RPL is intended to facilitate the functionalities of numerous link-layer protocols. These layers may be lossy or consumed with strongly constrained devices. The RPL can have alternative routes and adapt with the network conditions when there is no access to the default routes.

### 1.1. RPL Routing Protocol Overview

RPL was designed to support the vision of Wireless Sensor Networks (WSNs) and the Internet of Things (IoT) domains. RPL is also intended to be an interoperable and simple networking protocol to meet the requirements of resource-constrained devices and applications. RPL is a distance-vector routing protocol with its topology based on a Destination Oriented Directed Acyclic Graph (DODAG). The DODAG root is used to collect default routes to the internet and distribute them into other routing protocols. Each node in the topology is assigned a rank value that defines the position of the node with respect to the DODAG root. In addition, according to the specification of the RPL, each DODAG root node contains a version number. To revalidate the integrity of the DODAG root node and launch a global repair mechanism, a DODAG new version is formed by incrementing the version number that is determined by the root node only. The creation of the RPL network topology is maintained with five control messages, which are (1) DODAG Information Object (DIO), (2) DODAG Information Solicitation (DIS), (3) Destination Advertisement Object (DAO), (4) DAO Acknowledgement (DAO-ACK), and (5) Consistency Check (CC). Depending on the Objective Functions (OF), the RPL can nominate the optimal route to define the parent and neighbor node selection [5].

### 1.2. RPL Security

Secure routing protocols for data packets in IoT networks is a challenging issue due to the characteristics that are inherited from other networks [6]. Data packet routing in IoT-constrained devices suffer from potential security threats, and this has a considerable impact since it is related to the users [7]. Several RPL attacks occur through the activities of malicious nodes during the data packet routing among devices [8]. The RPL security has been extensively reviewed in [9,10,11]. Various kinds of RPL attacks have been analyzed, yet most of the studies have not concentrated on the mechanisms of secure RPL. In recent years, efforts have been made to design secure routing protocols for IoT-constrained devices. However, they all rely on conventional cryptographic functions, which drastically drain the resources of devices and impact the performance of the constrained devices likely to be used in IoT applications. The RPL characteristics, such as a lack of infrastructure, unreliable links, resource constraints, limited physical security, and dynamic topology, make them vulnerable and hard to protect against attacks. The data traffic density in certain nodes can lead to depleting batteries and resources faster than other nodes, which deactivates the routing data to the root node. Due to the billions of interconnected devices on the network, securing and protecting them from different forms of attacks creates a critical challenge. When these devices are vulnerable to attacks, such as rank attacks and version number attacks, users will feel that their data are insecure [7,8].

### 1.3. Research Contribution

RPL protocol security has been extensively studied because of the innumerable security threats to resource-constrained devices. The RPL rank and version number attacks, which are two types of RPL attacks, can have critical consequences for RPL networks. A rank attack affects the network performance, low Packet Delivery Ratio (PDR), and the delay and generation of non-optimal paths and loops. A version number attack affects the network performance due to increased overhead control, a low delivery of packet ratio, and a high end-to-end delay. The studies conducted on these attacks have several flaws, such as the following:

- several security defects and shortcomings regarding network performance and accuracy;
- multiple attacks in RPL networks are not supported;
- multiple RPL network topologies are not supported;
- they do not detect and mitigate the effects of both attacks in the RPL network; and
- they do not isolate the malicious nodes from legitimate nodes in the RPL network.

Therefore, there is a requirement for further research to handle the declared security problems for RPL routing protocols in IoT. Accordingly, this research work will extend our published previous work [12] that investigated in detail the existing research literature and research gaps of RPL attacks, concentrating on the rank attack and the version number attack. In this work, we propose a Secure RPL Routing Protocol (SRPL-RP), and in addition, our research will be based on the work presented in [13,14] by addressing their existing issues. The main contribution of this research is a combination of the research work in [13,14] and the addition of new features as follows:

- the addition of a timestamp threshold to verify the legitimacy of the sender nodes;
- a monitoring table that captures all information of the nodes in the RPL network topology;
- detection of both rank attack and version number attacks based on a comparison of ranks strategy;
- mitigation of the effects of both rank attacks and version number attacks by updating a monitoring table;
- isolation of both rank attacks and version number attacks by using a blacklist table to prevent malicious nodes from joining the RPL network and alerting other nodes to skip them; and
- the provision of multiple types of attacks (rank and version number attacks) in RPL networks, and support for different types of RPL network topologies.

### 1.4. Research Paper Organization

The research paper is organized as follows: in Section 2, we present the literature review of the recent studies related to RPL rank attacks and RPL version number attacks. In Section 3, we describe the proposed SRPL-RP along with its design, description, and implementation. In Section 4, we give an overview of the simulation setup and present the analysis of the results of the proposed protocol and existing countermeasures. In Section 5, we present the discussion, which demonstrates the security analysis of the proposed SRPL-RP and justifies that SRPL-RP can significantly provide better results than the existing countermeasures in terms of network performance and accuracy. Finally, our conclusions are provided in Section 6, which wraps up the research, achieved objectives, and future works.

## 2. Literature Review

This section will introduce the RPL attacks and their obstacles. We present the latest research concerning the RPL security.

### 2.1. RPL Rank Attack

A rank attack in the RPL network topology exposes the child nodes that have a deeper rank in the network. Then, the malicious nodes can change the method, in which the neighbor nodes can process their DODAG Information Object (DIO) messages. The rank attack can disrupt the data packet’s delivery path in the network topology. In a rank attack, the location of the parent node and neighbor nodes always attract the child nodes. Therefore, the malicious node advertises a fake rank node’s location in the RPL control messages or a fake route across the root node to deceive close nodes and attract them to forward their packets through it. For example, the child node’s deeper rank is reduced to be selected by the neighbor nodes as the parent node, to send data packets to the root node. Once the child node receives the data packets, then the child node drops the data packets.

Hence, the child node is treated as a malicious node. The rank attack has several effects, such as (1) un-optimized route formulation, (2) un-recognized formulated loop, (3) the RPL network topology never utilized the optimized routing, (4) when the malicious nodes increase, there will be a decrease in the PDR and small modification of the end-to-end delay, and (5) there will be an increase in the DIO messages due to the rapid changes in the network topology. Consequently, the network constrained merits are influenced, such as the energy consumption, delay, packet delivery ratio, and control overhead [15]. Unauthorized access by attackers or third parties to data routing in the RPL networks can make the RPL security a severe problem that shall be considered [16].

The sub-sections will give details and classify the RPL rank attack countermeasures.

#### RPL Rank Attack Countermeasures Classification

1 Classification-Based RPL Rank Attack Modification Techniques

The authors in [17] proposed and developed the Secure-RPL (SRPL) protocol. The malicious node(s) in the proposed protocol is blocked from better self-repositioning in the DODAG tree of the RPL network. The proposed protocol scans the number of times that the nodes’ rank values increase by enabling a threshold function to reduce the impact of the attack in the network. The evaluation results of the network performance indicated that the proposed protocol was efficient in protecting the RPL network. To overcome the overhead that existed in [18], Airehrour et al. [19] developed and proposed a Time-Based Trust-Aware RPL (SecTrust-RPL) to provide secure protection against rank attacks and Sybil attacks. This provides detection and isolation of the attacks with network performance optimization. Each node in the RPL network, in which its neighbor nodes have direct trust values and recommended trust values, computes a trustworthiness. Based on the evaluation results, the proposed protocol has better protection against rank attacks.

2 Classification-Based IDS

The authors in [20] designed a Specification-Based IDS. To detect attacks, this system uses Finite State Machine (FSM) transitions, and Monitoring Nodes (MN) are formed in the monitoring architecture. To detect a rank attack, the malicious nodes with lower ranks are scanned by the MN. The MN will suspect action changes of the valid rank and the fake rank of the malicious nodes. The information cross-checking of the MN will be started to detect the valid ranks. The study in [21] proposed a secure parent node selection scheme, in which, based on a threshold value, a legitimate node will be selected by the child nodes as their parent node. Every node in the RPL network decides the rank value that is advertised by the neighbor nodes based on the threshold between the maximum and average rank. If the rank value is too low, it will be selected as a parent node. The evaluation results of the scheme show that it is effective in decreasing the linking of the child nodes with the malicious nodes.

Althubaity et al. [22] designed an Authentication Rank and Routing Metric (ARM), which is a hybrid specification-based ID. The sink node in ARM is defined as a centralized module, while other nodes are defined as a distributed module. The centralized module works in the DIO message analysis and decision-making participation. On the other hand, the distributed module works by alerting the sink nodes regarding any changes that happened in the destination nodes. The evaluation results indicate that ARM safeguards the RPL network with a high accuracy rate. The researchers in [13] presented a Sink-Based Intrusion Detection System (SBIDS) to detect rank attacks in the RPL network. This system works by the rule of comparing the Node Current Rank (NCR) with the Node Parent Rank (NPR) and checking the minimum rank between their siblings. The evaluation results of SBIDS showed that it was effective in detecting rank attacks.

### 2.2. RPL Version Number Attack

The version number attack in the RPL network topology can illegitimately increment the root node’s DODAG version number by the malicious node when the DIO message is forwarded to its neighbor nodes to damage the network performance. When the neighbor nodes receive the DIO message that contains the incremented version number, the DODAG tree begins a new formulation, and the trickle timer is reset [23]. After that, the neighbor nodes will transmit the frequently updated version of the DIO messages to all nodes [24]. The version number attack has significant impacts, such as (1) the operation of the network is damaged; (2) the network control overhead is increased 18 times, which is unnecessary; (3) there will be routing loops in the data routing; (4) the network energy consumption is increased; and (5) the communication channels of the nodes have availability issues. In addition, packet delivery is lost, and the network delay is doubled [25].

#### RPL Version Number Attack Countermeasures Classification

The rank attacks countermeasures are classified into two categories, which are (1) modification techniques that can adjust or add the RPL standards, and can detect a limited number of attacks, and (2) Intrusion Detection Systems (IDS) that require nodes collaboration, and can detect multiple types of attacks [17].

1 Classification Based RPL Version Number Attack Modification Techniques

The study in [26] proposed and implemented a rank and version number authentication security measure scheme based on one-way hash chains called Version Number and Rank Authentication (VeRA). This provides security against internal attacks that broadcast an incremented version number or a higher rank in the DIO messages. The version number is checked for whether it was updated by the root node or not and whether the rank value of the parent node is illegitimately increasing or not. The evaluation results show the overhead time of the scheme. Perrey et al. [27] proposed and designed a Trust Anchor Interconnection Loop (TRAIL) scheme to overcome the obstacles in the former study [26] by analyzing the incompleteness of the rank authentication message. The sink node works as a trust anchor, and every node in the RPL network validates each rank value and drops invalid rank values.

The studies above that are used to detect the version number attacks can suffer from increased overheads. Therefore, to safeguard against the version number attacks, the authors in [28] proposed and developed a cooperative, distributed verification mechanism. The mechanism depends on checking the step phase and verification phase. The cooperative verification procedure works by allowing the receiving nodes to verify the neighbor node’s identity to determine whether the neighbor nodes have malicious behavior or not. The evaluation results showed that the control overhead was decreased and the mechanism was reliable.

To mitigate the effects of the version number attacks, the researchers in [29] proposed and designed a lightweight approach. Every node in the RPL network executes independent algorithms, in which the state of the nodes is not stored. The evaluation results indicated that the proposed scheme was lightweight and compatible with constrained devices. The research in [14] proposed and implemented lightweight techniques for version number attacks to consider the version number legitimate update. The elimination technique eliminates the malicious update influences of the version number. A trust mechanism is used by the shield technique, in which a change to the version number is required if the majority of the neighbor nodes that are close to the root node have a better rank. The evaluation results indicate that it is possible to mitigate the version number attack using these techniques.

2 Classification-Based IDS

Mayzaud et al. [30] proposed and developed a mechanism to detect and identify the malicious nodes that have illegitimately incremented version numbers based on the distributed monitoring architecture. This mechanism detects and monitors the operations of the node in the RPL network based on the monitored nodes (regular nodes) and the monitoring nodes, in which detection operations are performed. The evaluation results showed that the mechanism had a satisfying performance.

The literature review clarified that the RPL security has been widely studied due to the huge number of threats in the IoT. Researchers [31,32,33] developed many solutions for RPL rank attacks and version number attacks. These attacks need to be handled because of the trade-off between providing a safeguard against them and maintaining an efficient performance of the network in the IoT environment. The studies are effective in detecting these attacks, but they suffer from many flaws that have to be addressed. Based on the analysis in [12], we can observe that there are several parameters that can have an impact on the network performance and detection accuracy, such as (1) the RPL network topology type; (2) the number of nodes; and (3) the malicious node locations. Therefore, to support these observations, we propose a SRPL-RP to detect, mitigate, and isolate these attacks in the RPL network. In addition, this system supports different types of attacks with multiple types of RPL network topologies.

## 3. The Proposed Protocol

The proposed protocol is introduced in this section. The proposal and design of SRPL-RP is explained with its description, flow chart model, and implementation.

### 3.1. SRPL-RP Proposal

We present the proposed SRPL-RP to detect, mitigate, and isolate the attacks discussed in the previous section. The declared security issues for the RPL protocol can be handled by having the following features in the proposed protocol:

1. A timestamp threshold to verify the legitimacy of the sender nodes.
2. A monitoring table during the construction of DODAG that contains information regarding the nodes.
3. Detection of rank attacks and version number attacks.
4. Mitigation of the effects of rank attacks and version number attacks.
5. Isolation of rank and version number attacks.

The two sections below describe the protocol model flowchart and implementation, which are presented for consideration in this proposed protocol.

### 3.2. Attacker Model

In this section, the attacker model of the proposed protocol is introduced. The RPL network topology comprises one root node, multiple normal nodes, and some malicious nodes that are rank attacks and version number attacks. We are assuming that the root node cannot be exposed, and its ID is encrypted and cannot be violated [13]. The proposed protocol is safe from insider attacks using Elliptic Curve Cryptography (ECC) [34]. In RPL, the version number and rank are carried in a DIO message, and the version number is used as an indicator for the global repair operation. The DODAG root node is the only node that can change the version number. All the nodes in the RPL network topology begin exchanging control messages to rebuild the network topology after the root nodes change the version number.

While sending the DIO packet, malicious nodes attach their rank and version in the DIO packet. Subsequently, the attacker can exhaust the restricted drain and the limited resources of all the nodes in the RPL network, and this can lead to detrimental impacts on the network performance. The malicious nodes start their attacks by broadcasting a fake rank and version number during the cycle RPL trickle time. The version attacker is the one that changes the version number of nodes by incrementing their nodes, and a rank attacker is the one that falsely proposes the rank value to be chosen as a parent node. The nodes can spread their version and rank in the DODAG. While receiving the DIO packets from the malicious nodes (including rank and version), the current node changes their rank and version. Hence, they cannot determine the path to reach the root node.

### 3.3. SRPL-RP Description

This section depicts the details of the proposed protocol that detects, mitigates, and isolates the malicious nodes of both rank attacks and version number attacks. When a normal node receives a DIO control message, the protocol starts, and it consists of five phases:

Phase One: A timestamp is used to monitor and track the time that the DIO control messages are exchanged using the RPL trickle timer for synchronization. The difference in time between each DIO message has to not exceed a threshold value (which is calculated based on certain equations [31,35]). The time difference is registered as a timestamp and transmitted with the DIO message; thus, this helps in preventing malicious nodes. This timestamp is also used to determine the freshness of the DIO message throughout the process. If the time of the DIO message is above the threshold value, the DIO message will be discarded because it is indicated as malicious activity. In addition, if the time of the DIO message is less than the threshold value, then phase two is started.

Phase Two: If the DIO message has a lower value than the threshold value, the legitimacy of the sender node is verified by the receiver node by checking its ID. If it is invalid, the sender node will be discarded. If it is valid, the sender node will be added to a monitoring table (that is formulated during the DODAG construction), which captures information about the node, like the node ID, node rank, DIO message information, version number, etc. Hence, by using the monitoring table, the legitimacy of the nodes is verified, during which every valid node will be added to the monitoring table. Thus, when the receiver node checks the sender’s node ID, it will refer to this table to check if the sender’s ID exists in the monitoring table or not.

Phase Three: We combine the research work [13,14] and add new features to them, which are a monitoring table, blacklist table, and an alert function to detect, mitigate, and isolate both types of attack. Table 1 compares our work and the changes added to the work of [13,14]. If the DIO message of the sender node does not have a greater version number than the version number of the root node (assuming that the root node cannot be compromised), then it will be a case of rank attack detection and mitigation. If the DIO message of the sender node has a version number greater than the version number of the root node, then it will be the case of version number attack detection and mitigation.

**Figure 1:**
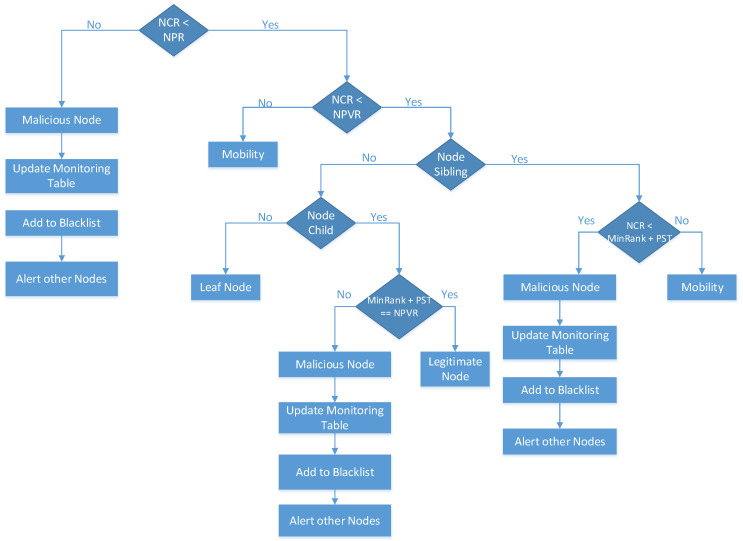
Protocol Model Flowchart, Phase Four.

Phase Four: Figure 1 shows the conditions for rank attack detection, mitigation, and isolation, which are added to the research [13]. If the Node Current Rank (NCR) is greater than the Node Parent Rank (NPR), then it is considered to be a malicious node. If the DIO control message of the malicious node is not discarded and is falsely verified as a legitimate node in the monitoring table for any reason, then the monitoring table will be updated to remove all information of the malicious node to restrict it from being a parent node. The malicious node will be added to the blacklist table (which is formulated during the DODAG construction), which captures all information of the malicious nodes to mitigate the effect of the malicious node from the network. The blacklist table contains the IDs of all malicious nodes that should not join the RPL network topology again because they were previously detected as malicious nodes. Then, an alert message will be sent to all the nodes in the network to notify them not to join this node in the future; thus, the malicious node is isolated from the network.

On the other hand, if the NCR is lower than the NPR, then the rank rule of the current node is compared with the rank rule of the previous rank. If the NCR is greater than the Node Previous Rank (NPVR), then it is considered a mobile node in the RPL network. When a node reaches its final destination, it does not change its rank, but it is stabilized concerning its neighboring nodes. However, if the NCR is lower than the NPVR, then the system checks whether the nodes are siblings. If the node does not have siblings, then the system checks whether they are child nodes. If the node is not a child, then it is a leaf node. In addition, if the nodes are children, then the minimum rank and Parent Switching Threshold (PST) is compared with the NPVR. If the (minimum rank + PST) is equal to the NPVR, then the node is legitimate and valid.

If (minimum rank + PST) is not equal to the NPVR, then it is considered a malicious node. The monitoring table will be updated to add the malicious node to the blacklist table. On the other hand, if the node has siblings, then the NCR is compared with the minimum rank and PST. If the NCR is lower than the (minimum rank − PST), then it is considered and detected as a malicious node. The monitoring table will be updated to add the malicious node to the blacklist table. However, if the NCR is greater than the (minimum rank − PST), then it is considered a mobile node in the RPL network.

**Figure 2:**
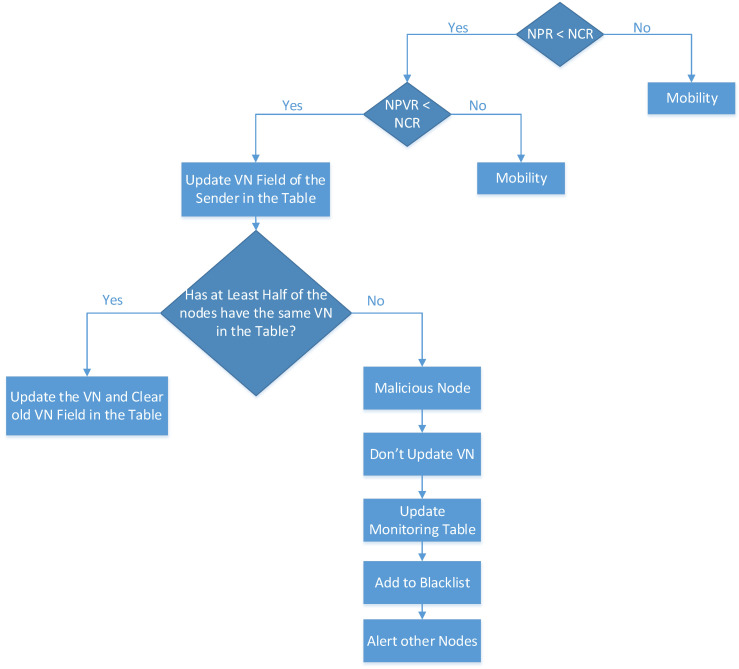
Protocol Model Flowchart, Phase Five.

Phase Five: Figure 2 shows the conditions for the version number attack detection, mitigation, and isolation, which is added to the research [14]. If the DIO message of the sender node has a greater version number than the version number in the root node, then the rank rule of the parent node is compared with the rank rule of the current node. If the NPR is greater than the NCR, then it is considered as a mobile node in the RPL network. However, if the NPR is lower than the NCR, then the rank rule of the previous node is compared with the rank rule of the current node. If the NPVR is greater than the NCR, then it is considered a mobile node in the RPL network. However, if the NPVR is lower than the NCR, then the version field of the sender node is updated in the neighbor list table.

The neighbor table list is formulated during DODAG construction, and it stores the information of the neighbor nodes and their version field. Then, the system checks whether half of the nodes have the same information of the version number in the neighbor list table. If half of the neighbor nodes in the neighbor list table have the same version number, then the version number of the sender node is updated and changed to the same majority version number in the table. This also clears the previous version number field. However, if half of the nodes do not share the same information of the version number field in the table, then it is considered a malicious node. The monitoring table will be updated to add the malicious node to the blacklist table.

### 3.4. SRPL-RP Implementation

Based on the creation of the rank attack and version number attack, a timestamp is attached to the DIO control messages. The timestamp is used to monitor and track the time of exchange of the DIO control messages. The time difference between DIO messages should be within a threshold value that is registered as a timestamp and transmitted with the DIO message. If the time of the DIO message is above the threshold value, the DIO message will be discarded because it is indicated as a malicious activity. In addition, if the time of the DIO message is less than the threshold value, the legitimacy of the sender node will be verified by the receiver node for more security by checking the ID of the sender nodes against the values in the monitoring table that was created during the establishment of the RPL DODAG by the root node. If it is valid, the node will be added to the monitoring table. After that, if the node version number in the DIO message is greater than the default version number in the root node, the rank attack will be checked. The rank value of the rank must be checked according to the comparison strategy.

#### 3.4.1. Rank and Version Number Attacks Detection

A node table is used to access the ranks of the parent, child, and neighbor nodes. The node needs first to satisfy the parent and child rank relationship. The parent should have a lower rank value compared to that of the child. Then, the NCR is compared with its NPR and NPVR. The node’s rank is comparatively evaluated against child and sibling rank, by following Algorithms A1 and A2 in the appendix section, as referred to in Figure 1 in Section 3. In Algorithm A1, if the minimum rank among sibling nodes that are deduced from minimum PST is greater than the NCR, then the node is considered a malicious one; otherwise, it is considered a legitimate node. Similarly, in Algorithm A2, if the minimum rank among child nodes that are summed together with PST is greater than or equal to the NCR, then the node is considered a malicious one; otherwise, it is considered as a legitimate node.

On the other hand, a version number attack is detected if the version number node is greater than the default root node’s version number (240). The NCR is compared with its NPR (the parent rank must be lower than the current rank). Similarly, the node rank is compared with its NPVR. If the NPVR is lower than the NPR, then the network is stabilized, and the version field of each node in a table needs to be checked (after receiving the DIO). Otherwise, the node needs to update its version number. If half of the neighbor nodes in the neighbor table list have the same version number, then the version number in the DIO message of the current node is updated. Then, the version number is changed to the same majority version number in the list by checking the condition (version != 240) in Algorithm A3 found in the appendix section, referred to in Figure 2 in Section 3.

#### 3.4.2. Rank and Version Number Attacks Mitigation

For mitigation purposes, in the version number attack, if a node has malicious behavior, then the malicious version number will behave as a legitimate node by updating its version number to the same one as in the neighbor list table. With this technique, nodes are prevented from being the attacker. At every DIO reception, the table will be updated as in Algorithm A4, found in the appendix section. In the rank attack, we set the attack status in the monitoring table to restrict the malicious node from being a parent node in Algorithm A5 found in the appendix section, referred to in Figure 1 and Figure 2 in Section 3. Hence, the mitigation mechanism occurs.

#### 3.4.3. Rank and Version Number Attacks Isolation

A threshold alert is attached to the DIO messages to implement the isolation feature of malicious nodes from the RPL network for supplemental security. The malicious nodes will be added in the blacklist table to send an alert message to other nodes in the RPL network to skip the malicious nodes. Hence, every node in the RPL network sends DIO messages to other nodes to prevent the malicious nodes from sending DIO messages as is depicted in Algorithm A6, found in the appendix section, and referred to in Figure 1 and Figure 2 in Section 3.

## 4. Simulation and Results Analysis

This section will present the simulation setup and performance parameters to simulate and measure the effectiveness of our proposed protocol.

### 4.1. Simulation Setup

To implement and measure the effectiveness of the proposed secure protocol, the Cooja simulator program on Contiki Operating System (Contiki OS 3.00) was used [36]. This is a networking system and a multitasking operating system for IoT devices. Hence, this system was used for creating different simulations in this research paper. We conducted three types of topologies to analyze the security effectiveness of the proposed protocol and the network performance: grid-center topology, Grid-random topology, and random topology. The nodes were placed in a 100 m x 100 m area, and each node was distributed in a transmission range of 50 m that maintained the linkage between nodes and the interference range of 100 m based on the Unit Disk Graph Medium (UDGM)-Distance Loss model (link failure model). These parameters are the default settings of the Cooja simulator. The network topology can be deployed in E-applications [37]. Table 2 shows a summary of the simulation model.

### 4.2. Results Analysis

This section will present an analysis of the proposed SRPL-RP. We tested the proposed SRPL-RP in the grid-center network topology, grid-random network topology, and random network topology. We ran and repeated the simulations 60 times for the three types of topologies at different time stages: the network convergence, the network stability, and the network at the end of the simulation. This measures the changes in the security accuracy and the network performance levels of the proposed protocol concerning times of 3 min, 15 min, 30 min, 45 min, and 60 min. The extracted results of the proposed SRPL-RP were used for comparison with the existing countermeasures, which were the Standard RPL with Attacks, SBIDS [13], and RPL+Shield [14], to measure the effectiveness and performance.

#### 4.2.1. The Network Performance Results and Comparison

In this sub-section, we present the performance of our proposed SRPL-RP for rank attacks and version number attacks. We compared the results with the standard RPL under rank attacks and version number attacks, as well as SBIDS [13] and RPL+Shield [14].

##### Network Performance Results

The simulation results show the network performance parameters of SRPL-RP, Standard RPL with attacks, SBIDS [13], and RPL+Shield [14] in the three topologies and time stages.

**Figure 3:**
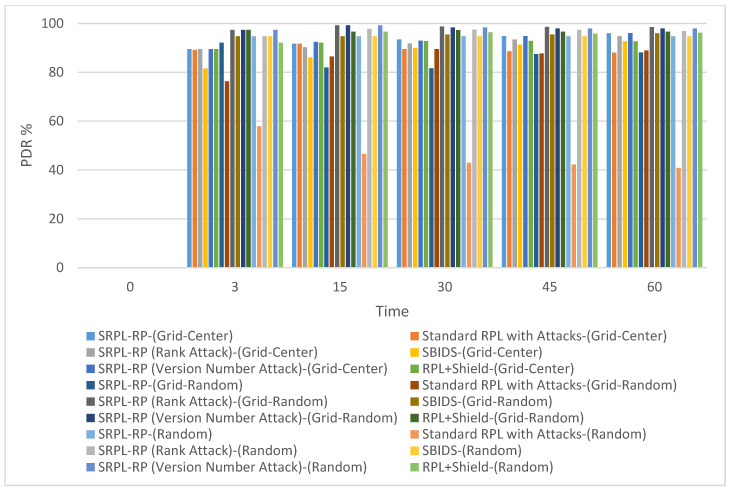
Packet Delivery Ratio Comparison in Three Topologies.

**Figure 4:**
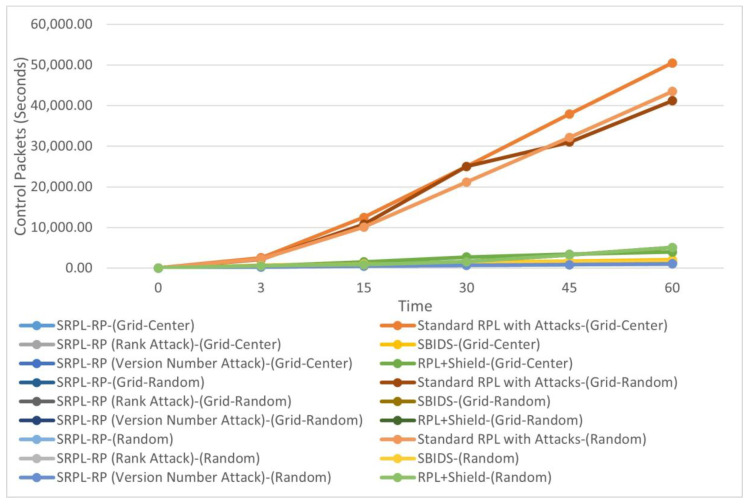
Control Message Comparison in Three Topologies.

**Figure 5:**
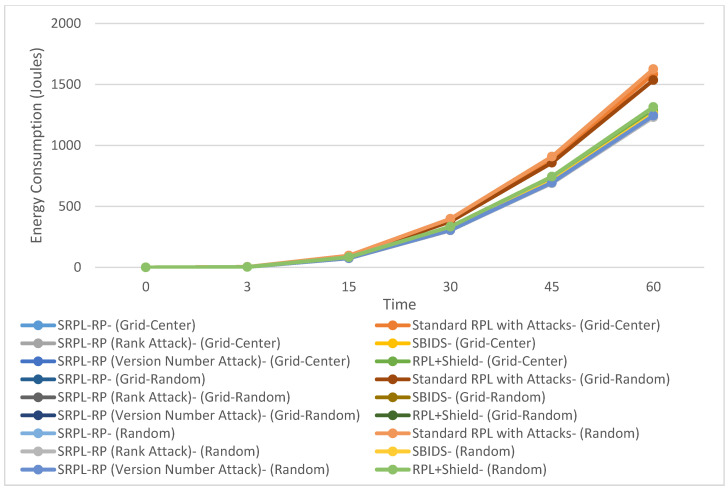
Average Energy Consumption in Three Topologies.

Figure 3 shows the PDR results of SRPL-RP compared with Standard RPL with Attacks, SBIDS [13], and RPL+Shield [14] in the three topologies. Figure 4 shows the PDR results in the grid-random topology. Figure 5 shows the results in the random topology. The results of the analysis and the comparison linked to the chart are presented in Table 3, Table 4 and Table 5, respectively. We noticed that the results show that the SRPL-RP had a higher PDR in the grid-center topology than in other topologies compared with Standard RPL with Attacks. In addition, the SRPL-RP (Rank Attack) in the grid-random topology had the highest PDR and performed better than in the other topologies compared with SBIDS [13]. The PDR was higher and better in the random topology than in other types of topologies for SRPL-RP (Version Number Attack) compared with the RPL+Shield [14].

Figure 4 shows the Control Message results of SRPL-RP compared with Standard RPL with Attacks, SBIDS [13] and RPL+Shield [14] in the three topologies. The results of the analysis and comparison linked to the chart are presented in Table 6, Table 7 and Table 8, respectively. These results show that our proposed protocol SRPL-RP in the random topology had the highest performance in reducing the redundant amount of produced control messages compared with Standard RPL with Attacks, SBIDS [13], and RPL+Shield [14], which produced more generated control messages.

Figure 5 shows the Average Energy Consumption of SRPL-RP compared with Standard RPL with Attacks, SBIDS [13], and RPL+Shield [14] in the three topologies. The results of the analysis and a comparison linked to the chart are presented in Table 9, Table 10 and Table 11, respectively. These results show that our proposed protocol SRPL-RP reduced the average energy consumption by up to 60% and was much lower in the random topology than in other topologies compared with Standard RPL with Attacks, SBIDS [13], and RPL+Shield [14].

#### 4.2.2. Accuracy Results

**Figure 6:**
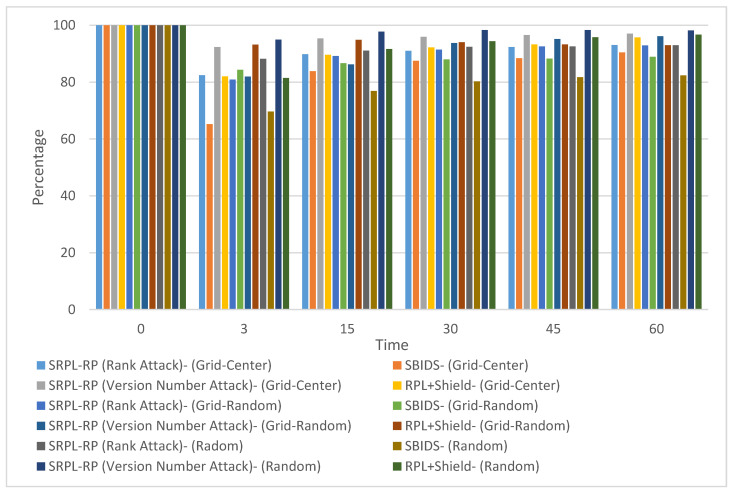
Accuracy Rate (AR) Comparison Three Topologies.

In this sub-section, we divided the proposed SRPL-RP into two groups based on the rank attack and version number attack to compare and evaluate them with SBIDS [13] (which provides detection against the rank attack) and RPL+Shield [14] (which provides mitigation against version number attack). We analyzed the effectiveness of the proposed SRPL-RP (Rank Attack) in detecting and mitigating the attack by classifying legitimate nodes and malicious nodes in the three types of topologies and comparing the results with SBIDS [13] and RPL+Shield [14]. Figure 6 shows the Accuracy Rate (AR) of SRPL-RP (Rank Attack) and SRPL-RP (Version Number Attack) compared with SBIDS [13] and RPL+Shield [14] in the three topologies. The results of the analysis and comparison linked to the chart are presented in Table 12, Table 13 and Table 14, respectively.

These results show that our proposed protocol SRPL-RP (Rank Attack) in the grid-center topology had the highest AR among other topologies compared with SBIDS [13]. The proposed protocol in the grid-random topology had the highest True Negative (TN) accuracy and the lowest False Positive (FP) accuracy, among other topologies compared with SBIDS [13]. The proposed protocol in the grid-center topology had the lowest False Negative (FN) accuracy and the highest True Positive (TP) accuracy, among other topologies compared with SBIDS [13]. These results show that our proposed protocol SRPL-RP (Version Number Attack) in the random topology had the highest AR among other topologies compared with RPL+Shield [14]. The proposed protocol in the grid-center topology had the highest TN accuracy and the lowest FP accuracy among other topologies compared with RPL+Shield [14]. The proposed protocol in the random topology had the lowest FN accuracy and the highest TP accuracy among other topologies compared with RPL+Shield [14].

## 5. Discussion

In this section, we demonstrate the security analysis of the proposed SRPL-RP and present the research findings and compare them with existing countermeasures to justify the effectiveness in terms of the network performance and the detection and mitigation accuracy.

### 5.1. Network Performance Discussion

From the analysis of the results in Section 4, we found that SRPL-RP in the grid-center topology, SRPL-RP (Rank Attack) in the grid-random topology, and SRPL-RP (Version Number Attack) in the random topology had the highest PDR and the best performance among other topologies compared with Standard RPL with Attacks, SBIDS [13], and RPL+Shield [14]. On the other hand, the effects of attacks in Standard RPL with Attacks was almost doubled in the random topology, causing routing errors with the majority of packets lost at the routing layer due to non-existing routes. Even though the SBIDS [13] provided detection against rank attack, it still had a lower impact by providing better PDR compared with our SRPL-RP (Rank Attack), especially in the grid-center topology, where the effect of rank attacks was almost doubled.

The effect of the version number attack in RPL+Shield [14] was almost tripled in the grid-center topology, even though this provided mitigation of the attack compared with our SRPL-RP (Version Number Attack). These results show that the best average results for PDR can be extracted in the grid-center topology. This is because the nodes are placed in uniform distribution and densities, and this topology ensures that each node can reach only its vertical and horizontal neighbors during the simulation. Thus, this influences the RPL network and the number of parent nodes and child nodes that are created by the DODAG, in which a smaller number of parent nodes serve more child nodes.

In the grid-random topology and random topology, each node may have more parent nodes. Hence, the parent nodes allow most of their child nodes to listen to the control messages. Thus, the DODAG of the RPL network can be constructed with more control messages. Therefore, PDR mainly depends on the node distribution and network topology; thus, nodes that have more child nodes have a higher probability of having a higher PDR. When the malicious nodes are closer to the root node, they can be easily detected by the proposed protocol. This is because, when the malicious nodes are far from the root node, it may take longer for the root node to realize that there is a change in the network. As a result, it becomes harder to be detected, and by the time the changes are recognized in the network by the root node, the rest of the legitimate nodes can be affected by the attack.

We found that SRPL-RP in the random topology, SRPL-RP (Rank Attack) in the random topology, and SRPL-RP (Version Number Attack) in the random topology had the lowest and best performance in reducing the redundant amount of produced control messages compared with other topologies and compared with Standard RPL with Attacks, SBIDS [13], and RPL+Shield [14]. On the other hand, the effects of the attacks in Standard RPL with Attacks were higher in the grid-center topology. In addition, the number of generated control messages in SBIDS [13] is higher in the grid-center topology. Additionally, RPL+Shield [14] had more generated control messages even after applying the mitigation mechanism, especially in the random topology.

This shows that the best average results for a control message were extracted in the random topology. This is due to the nature of topologies in which the malicious nodes spread in a random topology faster than in the grid-center and grid-random topologies that have a unified nature. This affects the number of parents and child nodes that create the DODAG and that have more parent nodes in random placements, in which the parent nodes allow fewer of their child nodes to listen to the control messages. Thus, the DODAG of the RPL network can be constructed with more control messages. Hence, the proposed SRPL-RP can reduce the effect of excess generated control messages and successfully mitigate the effect of the attacks. Thus, it prevents the malicious nodes from rebuilding the DODAG with higher parent nodes, and fewer control messages will be generated.

We found that SRPL-RP in the random topology, SRPL-RP (Rank Attack) in the random topology, and SRPL-RP (Version Number Attack) in the random topology had the lowest and the best performance for the average energy consumption compared with other topologies and with Standard RPL with Attacks, SBIDS [13], and RPL+Shield [14]. On the other hand, the effects of attacks in Standard RPL with Attacks were higher in the random topology for the average energy consumption. These were also higher in SBIDS [13] in the grid-center topology. At the same time, the effects were higher in both the grid-random topology and the random topology compared with the grid-center topology of RPL+Shield [14] even after applying the mitigation mechanism. This shows that the best average results for the average energy consumption were extracted in the random topology. This is because there will be fewer paths among nodes owing to the impact of SRPL-RP, which results in fewer packets lost and generates fewer control messages. The average energy consumption was greater in the random topology compared with the other topologies and this was because of both attacks and because there existed longer paths among nodes. Thus, the majority of packets lost at the routing layer was due to routing errors caused by both attacks that made most of the nodes drop their packets, which consumed a great deal of energy.

### 5.2. Accuracy Discussion

From the analysis of the results in Section 4, we found that SRPL-RP in the grid-random topology, SRPL-RP (Rank Attack) in the grid-random topology, and SRPL-RP (Version Number Attack) in the grid-center topology had the highest TN accuracy and the lowest FP accuracy among other topologies compared with SBIDS [13] and RPL+Shield [14]. This indicates that, in the case of TN, the percentages of the total number of malicious nodes that were correctly identified as attacking nodes were: 97.65%, 98.04%, and 98.04% for SRPL-RP, SRPL-RP (Rank Attack), and SRPL-RP (Version Number Attack), respectively. On the other hand, in the case of FP, the percentages of the total number of malicious nodes that were falsely identified as legitimate nodes were: 2.35%, 1.96%, and 1.89% for SRPL-RP, SRPL-RP (Rank Attack), and SRPL-RP (Version Number Attack), respectively. These are very small numbers compared with SBIDS [13], which had 7.04%, and RPL+Shield [14], which had 7.61%.

We found that SRPL-RP in the grid-center topology, SRPL-RP (Rank Attack) in the grid-center topology, and SRPL-RP (Version Number Attack) in the random topology had the lowest FN accuracy and the highest TP accuracy among other topologies compared with SBIDS [13] and RPL+Shield [14]. This indicates that in FN, the percentages of the total number of legitimate nodes that were falsely identified as the malicious node were: 5.33%, 11.22%, and 1.35% for SRPL-RP, SRPL-RP (Rank Attack), and SRPL-RP (Version Number Attack), respectively. These are very small numbers compared with SBIDS [13] and RPL+Shield [14]. In TP, the percentages of the total number of legitimate nodes that were not influenced by the proposed protocol were: 94.67%, 88.78%, and 98.65% for SRPL-RP, SRPL-RP (Rank Attack), and SRPL-RP (Version Number Attack), respectively. We found that SRPL-RP in the grid-center topology, SRPL-RP (Rank Attack) in the grid-center topology, and SRPL-RP (Version Number Attack) in the random topology had the highest AR among other topologies compared with SBIDS [13] and RPL+Shield [14]. This indicates that the percentages of the total accuracy metrics were: 95.62%, 93.05%, and 98.30% for SRPL-RP, SRPL-RP (Rank Attack), and SRPL-RP (Version Number Attack), respectively, compared with SBIDS [13] and RPL+Shield [14].

The above analysis and discussion clarify that the proposed SRPL-RP was better in comparison with existing countermeasures in terms of the network performance and detection and mitigation accuracy. On the basis of the comparison in Table 15 of studies in the literature and the proposed SRPL-RP, we demonstrated that the proposed SRPL-RP can provide better functionalities, better network performance, and better detection accuracy. In addition, SRPL-RP supports against multiple attacks at the same time on the network. The effectiveness of the proposed SRPL-RP in terms of the network performance was better: in the grid-center topology for PDR, the best result obtained was 98.48%. In the random topology for the control message value, the best result obtained was 991 packets/s. In the random topology for average energy consumption, the best result obtained was 1231.778 joules. However, the effectiveness of the proposed SRPL-RP in terms of the accuracy was better: in the random topology of SRPL-RP (Version Number Attack) for AR, the best result obtained was 98.17% for the aforementioned reasons. The reason for this is that the proposed SRPL-RP can provide verification of the sender nodes by using the threshold. After the detection occurs, mitigation and isolation features can be applied to cope with the severe effects of both attacks.

To achieve supplemental security, a blacklist table with a threshold alert was implemented to isolate and alert all other nodes to skip the malicious nodes from the RPL network. Therefore, the proposed SRPL-RP can assist in the development and in the reduction of the risks of RPL network security. An improved and higher safeguard can be provided against these two attacks, while providing efficient services and boosting user confidence.

## 6. Conclusions

Data packets addressing and routing among IoT-constrained devices are issues due to the necessity of developing integrated protocols for data packet routing across different RPL networks. Several RPL attacks occur through the activities of malicious nodes during data packet routing among devices. This research studied the latest literature that focused on rank attacks and version number attacks. Considering the research gap, we found that the recent studies did not support multiple attacks in RPL networks for measuring the effectiveness of their proposals. They did not detect and mitigate the effects of both attacks in the RPL networks.

This research provided a proposal and implementation of the SRPL-RPL protocol, which can address the current flaws in the existing studies by limiting the impact of these attacks. According to the simulation results in the analysis, the proposed SRPL-RP is more secure and more efficient in terms of the network performance and accuracy. SRPL-RP was shown to provide a higher PDR and a lower control message value compared with the existing countermeasures in all types of network topologies. In addition, this protocol demonstrated more than a 95% accuracy in all kinds of network topologies.

## 7. Future Work

We aim to implement the feature of mobile nodes in the RPL network to safeguard these nodes against attacks. Hence, they have the ability to be scalable, communicating more efficiently and covering large networks such as smart city networks.

## Figures and Tables

**Table 1:** Comparison of our Work and the Work of [13,14].

Work/Feature	Verification of Nodes	Comparison of Rank Strategy (Detection)	Updating Version Number (Mitigation)	Monitoring Table (Mitigation)	Blacklist Table (Isolation)
Our work	Yes	Yes	Yes	Yes	Yes
Work [13]	No	Yes	No	No	No
Work [14]	No	No	Yes	No	No

**Table 2:** The Simulation Model Parameters.

Parameter	Value
Simulator	Cooja 3.0
Node Type	Wismote
Number of Nodes	20 with 1 root node, 14 normal nodes
Number of Malicious Nodes	5: 4 rank attacker nodes, 1 version number attacker node
Routing Protocol	RPL Protocol
Area	100 m * 100 m
Simulation Time	60 min
Transmission Range	50 m
Interference Range	100 m
Packet Send Interval	60 s
Data Packet Size	127 Bytes
Topology	Grid-Center, Grid Random, Random

**Table 3:** Packet Delivery Ratio Comparison in Grid-Center Topology.

Protocol/Time	SRPL-RP	RPL + Attacks	SRPL-RP (Rank)	SBIDS [13]	SRPL-RP (Version Number Attack)	RRPL + Shield [14]
Convergence Time	89.47%	89.19%	89.47%	81.58%	89.47%	89.47%
Stability Stage	93.43%	89.47%	91.83%	90.02%	92.92%	92.74%
Final Stage	95.99%	88.04%	94.82%	92.69%	96.07%	92.68%

SRPL-RP (Secure RPL Routing Protocol); RPL (Routing Protocol for Low Power and Lossy Networks); SBIDS (Sink-Based Intrusion Detection Systems).

**Table 4:** Packet Delivery Ratio Comparison in Grid-Random Topology.

Protocol/Time	SRPL-RP	RPL + Attacks	SRPL-RP (Rank)	SBIDS [13]	SRPL-RP (Version Number Attack)	RRPL + Shield [14]
Convergence Time	92.11%	76.32%	97.37%	94.74%	97.37%	97.37%
Stability Stage	81.67%	89.47%	98.73%	95.46	98.37%	97.28%
Final Stage	88.12%	89.93%	98.48%	95.99%	97.95%	96.61%

**Table 5:** Packet Delivery Ratio Comparison in Random Topology.

Protocol/Time	SRPL-RP	RPL + Attacks	SRPL-RP (Rank)	SBIDS [13]	SRPL-RP (Version Number Attack)	RRPL + Shield [14]
Convergence Time	94.74%	57.89%	94.74%	94.74%	97.37%	92.11%
Stability Stage	94.74%	43.01%	97.46%	94.74%	98.37%	96.37%
Final Stage	94.74%	40.82%	96.88%	94.74%	97.95%	96.24%

**Table 6:** Control Message Comparison in Grid-Center Topology.

Protocol/Time	SRPL-RP	RPL + Attacks	SRPL-RP (Rank)	SBIDS [13]	SRPL-RP (Version Number Attack)	RRPL + Shield [14]
Convergence Time	259 packets/s	2525 packets/s	267 packets/s	245 packets/s	364 packets/s	501 packets/s
Stability Stage	867 packets/s	25,008 packets/s	782 packets/s	1414 packets/s	1150 packets/s	2700 packets/s
Final Stage	1332 packets/s	50,462 packets/s	1180 packets/sond	2015 packets/s	1543 packets/s	3964 packets/s

**Table 7:** Control Message Comparison in Grid-Random Topology.

Protocol/Time	SRPL-RP	RPL + Attacks	SRPL-RP (Rank)	SBIDS [13]	SRPL-RP (Version Number Attack)	RRPL + Shield [14]
Convergence Time	414 packets/s	2146 packets/s	430 packets/s	619 packets/s	275 packets/s	555 packets/s
Stability Stage	1107 packets/s	25,008 packets/s	877 packets/s	1104 packets/s	689 packets/s	1570 packets/s
Final Stage	1468 packets/s	41,160 packets/s	1363 packets/s	1479 packets/s	1072 packets/s	5045 packets/s

**Table 8:** Control Message Comparison in Random Topology.

Protocol/Time	SRPL-RP	RPL + Attacks	SRPL-RP (Rank)	SBIDS [13]	SRPL-RP (Version Number Attack)	RRPL + Shield [14]
Convergence Time	255 packets/s	2247 packets/s	255 packets/s	630 packets/s	297 packets/s	555 packets/s
Stability Stage	658 packets/s	21,167 packets/s	658 packets/s	1272 packets/s	690 packets/s	1570 packets/s
Final Stage	991 packets/s	43,481 packets/s	991 packets/s	1676 packets/s	1095 packets/s	5045 packets/s

**Table 9:** Average Energy Consumption Comparison in Grid-Center Topology.

Protocol/Time	SRPL-RP	RPL + Attacks	SRPL-RP (Rank)	SBIDS [13]	SRPL-RP (Version Number Attack)	RRPL + Shield [14]
Convergence Time	259 joules	2525 joules	2.927 joules	3.084 joules	2.931 joules	3.236 joules
Stability Stage	867 joules	25,008 joules	309.474 joules	314.903 joules	311.687 joules	325.414 joules
Final Stage	1332 joules	50,462 joules	1258.783 joules	1276.162 joules	1263.291 joules	1287.982 joules

**Table 10:** Average Energy Consumption Comparison in Grid-Random Topology.

Protocol/Time	SRPL-RP	RPL + Attacks	SRPL-RP (Rank)	SBIDS [13]	SRPL-RP (Version Number Attack)	RRPL + Shield [14]
Convergence Time	414 joules	2146 joules	2.827 joules	2.973 joules	2.975 joules	3.326 joules
Stability Stage	1107 joules	25,008 joules	303.417 joules	309.661 joules	309.380 joules	333.502 joules
Final Stage	1468 joules	41,160 joules	1237.753 joules	1255.469 joules	1254.235 joules	1314.884 joules

**Table 11:** Average Energy Consumption Comparison in Random Topology.

Protocol/Time	SRPL-RP	RPL + Attacks	SRPL-RP (Rank)	SBIDS [13]	SRPL-RP (Version Number Attack)	RRPL + Shield [14]
Convergence Time	255 joules	2247 joules	2.939 joules	3.005 joules	2.876 joules	3.326 joules
Stability Stage	658 joules	21,167 joules	304.300 joules	311.054 joules	305.617 joules	333.502 joules
Final Stage	991 joules	43,481 joules	1231.778 joules	1259.908 joules	1244.819 joules	1314.884 joules

**Table 12:** Accuracy Rate (AR) Comparison Grid-Center Topology.

Protocol/Time	SRPL-RP (Rank)	SBIDS [13]	SRPL-RP (Version Number Attack)	RRPL + Shield [14]
Convergence Time	0%	0%	0%	0%
Stability Stage	91.00%	87.50%	95.93%	92.16%
Final Stage	93.05%	90.40%	97.03%	95.70%

**Table 13:** Accuracy Rate (AR) Comparison Grid-Random Topology.

Protocol/Time	SRPL-RP (Rank)	SBIDS [13]	SRPL-RP (Version Number Attack)	RRPL + Shield [14]
Convergence Time	0%	0%	0%	0%
Stability Stage	91.41%	88.00%	93.73%	94.04%
Final Stage	92.88%	88.90%	96.14%	92.93%

**Table 14:** Accuracy Rate (AR) Comparison Random Topology.

Protocol/Time	SRPL-RP (Rank)	SBIDS [13]	SRPL-RP (Version Number Attack)	RRPL + Shield [14]
Convergence Time	0%	0%	0%	0%
Stability Stage	92.41%	80.27%	98.17%	94.40%
Final Stage	92.97%	82.34%	98.30%	96.68%

**Table 15:** Comparison among Studies in Literature Review and our Proposed Protocol.

Study/Parameters	Support Multiple Attacks	Support Multiple Topologies	PDR	Control Message Overhead	Average Energy Consumption	AR
SRPL [18]	Yes	No	83%	1550	4320 joules	-
SecTrust-RPL [19]	Yes	No	80%	-	-	-
Specification-Based IDS [20]	Yes	No	-	-	-	-
Secure Parent Node Selection Scheme [21]	No	No	-	-	-	-
ARM [22]	No	No	-	-	3560.796 joules	60%
SBIDS [13]	No	No	95.99%	1479	1276.162 joules	90.40%
VeRA [26]	Yes	No	-	-	-	-
TRAIL [27]	No	No	-	-	-	-
Distributed and Cooperative Verification Mechanism [28]	No	No	97%	1500	-	-
Lightweight Defense Approach [29]	Yes	No	-	-	-	-
Lightweight Mitigation Techniques [14]	No	Yes	92.68%	5045	1314.884 joules	92.93%
Distributed Monitoring Strategy [30]	No	No	-	-	-	-
Our Proposed SRPL-RP	Yes	Yes	98.48%	991 packets/s	1231.778 joules	98.30%

## Abbreviations

**Term**	**Detail**
AR	Accuracy Rate
CC	Consistency Check
D2D	Device-to-Device
DAO	Destination Advertisement Object
DAO-ACK	DAO Acknowledgement
DIO	DODAG Information Object
DIS	DODAG Information Solicitation
DODAG	Destination Oriented Directed Acyclic Graph
ECC	Elliptic Curve Cryptography
FN	False Negative
FP	False Positive
IDS	Intrusion Detection Systems
IoT	Internet of Things
NCR	Node Current Rank
NPR	Node Parent Rank
NPVR	Node Previous Rank
Version Number	the version number of DODAG root node used for revalidation
OF	Objective Functions
PDR	Packet Delivery Ratio
PST	Parent Switching Threshold
Rank	Position of the node with respect to the sink
RPL	Routing Protocol for Low Power and Lossy Networks
SRPL-RP	Secure RPL Routing Protocol
TN	True Negative
TP	True Positive
UDGM	Unit Disk Graph Medium

## Appendix A

**Algorithm A1.** Evaluation of Node Current’s Rank and Node Sibling’s Rank	
**1:**	**Begin**
**2:**	**input:** node_id
**3:**	**input:** min_sibling_rank
**4:**	**input:** node_current_rank
**5:**	**input:** parent_threshold_divisor
**6:**	**input:** min_pst
**7:**	**input:** threshold
**8:**	**set** min_pst = (min_sibling_rank – parent_threshold_divisor)
**9:**	**if** node_current_rank < min_pst **then**
**10:**	**set** threshold[node_id] = 5
**11:**	**else**
**12:**	**set** threshold[node_id] = 4
**13:**	**End if**
**14:**	**End**

**Algorithm A2.** Evaluation of Node Current’s Rank and Node Child’s Rank	
**1:**	**Begin**
**2:**	**input:** node_id
**3:**	**input:** min_child_rank
**4:**	**input:** node_current_rank
**5:**	**input:** parent_threshold_divisor
**6:**	**input:** minch_pst
**7:**	**input:** threshold
**8:**	**set** minch_pst = (min_child_rank + parent_threshold_divisor)
**9:**	**if** node_current_rank <= minch_pst **then**
**10:**	**set** threshold[node_id] = 7
**11:**	**else**
**12:**	**set** threshold[node_id] = 6
**13:**	**End if**
**14:**	**End**

**Algorithm A3.** Checking the Condition of the Initial Default Version Number	
**1:**	** Begin**
**2:**	** input:** version
**3:**	** input:** neighbor_1_current_rank
**4:**	** input:** neighbor_1_previous_rank
**5:**	** input:** neighbor_1_version
**6:**	** input:** number
**7:**	** input:** neighbor_1
**8:**	** input:** neighbor_1_id
**9:**	** input:** neighbor
**10:**	** input:** neighbor_table_head
**11:**	** input:** neighbor_table_next
**12:**	** input:** n
**13:**	** input:** m
**14:**	** input:** p
**15:**	** if** version != 240 //DEFAULT VERSION(DODAG) = 240
**16:**	**for** neighbor_1 = neighbor_table_head; neighbor_1 != null; neighbor_1 = neighbor_table_next **then**
**17:**	**set** number ++
**18:**	**set** neighbor_1_id = address
**19:**	**set** neighbor[number] = neighbor_1_id
**20:**	**set** n[neighbor[number] = neighbor_1_current_rank
**21:**	**set** m[neighbor[number] = neighbor_1_previous_rank
**22:**	**set** p[neighbor[number] = neighbor_1_version
**23:**	** End for**
**24:**	** End if**
**25:**	** End**

**Algorithm A4.** Version Number Attack Mitigation	
**1:**	** Begin**
**2:**	**input:** version_count
**3:**	**input:** divisor
**4:**	**input:** version
**5:**	**input:** ver
**6:**	**input:** j
**7:**	**input:** threshold_table
**8:**	**input:** node_id
**9:**	**if** version_count >= divisor **then**
**10:**	**set** version = ver[j] //updating version number
**11:**	**set** threshold_table[node_id] = 2
**12:**	** else**
**13:**	**set** threshod_table[node_id] = 3
**14:**	** End if**
**15:**	** End**

**Algorithm A5.** Rank Attack Mitigation	
**1:**	**input:** preferred_parent1
**2:**	**input:** preferred_parent2
**3:**	**input:** preferred_parent1_status
**4:**	**input:** preferred_parent2_status
**5:**	**input:** parent1_metric
**6:**	**input:** parent2_metric
**7:**	**input**: p1
**8:**	**input**: p2
**9:**	**if** preferred_parent1 == 1 || preferred_parent2 == 1 **then**
**10:**	**if** parent1_metric < parent2_metric **then**
**11:**	**set** p1
**12:**	**else**
**13:**	**set** p2
**14:**	**End if**
**15:**	** else**
**16:**	**if** preferred_parent1_status != 1 && preferred_parent2_status != 1 **then**
**17:**	**if** parent1_metric < parent2_metric **then**
**18:**	**set** p1
**19:**	** else**
**20**	**set** p2
**21**	** End if**
**22**	** End if**
**23**	** End if**
**24**	** End**

**Algorithm A6.** Attacks Isolation	
**1:**	**input:** node_id
**2:**	**input:** attack_status
**3:**	**input:** alert
**4:**	**if** attack_status[node_id] == 0 **then**
**5:**	**set** alert: (legitimate node, node_id)
**6:**	**else**
**7:**	**if** attack_status[node_id] == 1 **then**
**8:**	**set** alert: (malicious node, node_id)
**9**	**End if**
**10:**	**End if**
**11:**	**End**
